# Supplementary material for: Splice-Junction-Based Mapping of Alternative Isoforms in the Human Proteome
Source: Cell Rep. Author manuscript; Available in PMC 2020 Jan 15. (PMC6961840; doi:10.1016/j.celrep.2019.11.026)
Supplement: 2 [file NIHMS1546469-supplement-2.zip › DF1/PXD010154/Esophagus/Esophagus_30_HMGB1_ENILACPLVMLR.pdf]

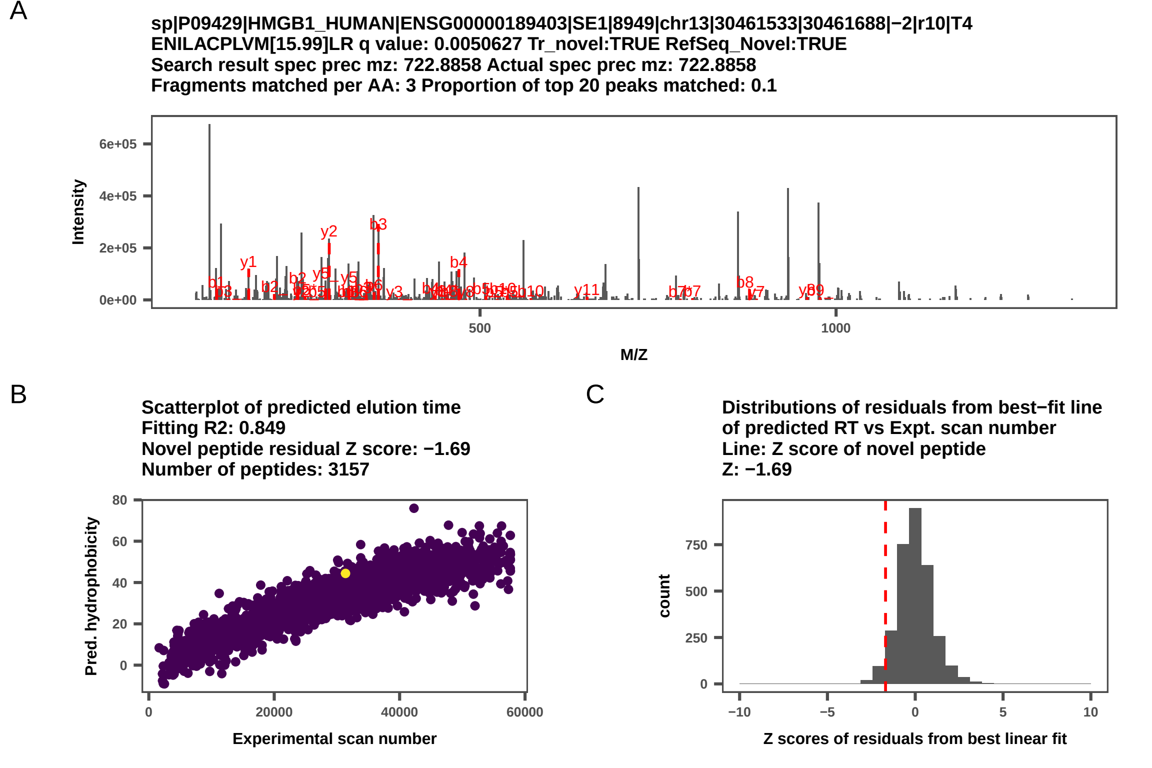
A
sp|P09429|HMGB1_HUMAN|ENSG00000189403|SE1|8949|chr13|30461533|30461688|−2|r10|T4
ENILACPLVM[15.99]LR q value: 0.0050627 Tr_novel:TRUE RefSeq_Novel:TRUE
Search result spec prec mz: 722.8858 Actual spec prec mz: 722.8858
Fragments matched per AA: 3 Proportion of top 20 peaks matched: 0.1
Intensity
6e+05
4e+05
2e+05
0e+00
500
1000
M/Z
y1
y2
b3
b4
b8
y11
B
Scatterplot of predicted elution time
Fitting R2: 0.849
Novel peptide residual Z score: −1.69
Number of peptides: 3157
Pred. hydrophobicity
80
60
40
20
0
0
20000
40000
60000
Experimental scan number
C
Distributions of residuals from best−fit line
of predicted RT vs Expt. scan number
Line: Z score of novel peptide
Z: −1.69
count
750
500
250
0
−10
−5
0
5
10
Z scores of residuals from best linear fit
